# Staged Management of Congenital Chylothorax With Hydrops Fetalis: An Insight Into EXIT Related Procedures

**DOI:** 10.3389/fped.2021.633051

**Published:** 2021-02-17

**Authors:** Hung-Lin Tai, Tze Yee Diane Mok, An-Shine Chao, Shih-Ming Chu, Reyin Lien

**Affiliations:** ^1^Department of Pediatrics, Saint Paul's Hospital, Taoyuan City, Taiwan; ^2^Division of Neonatalogy, Department of Pediatrics, Chang Gung Memorial Hospital, Taoyuan City, Taiwan; ^3^New Taipei Municipal TuCheng Hospital (Built and Operated by Chang Gung Medical Foundation), New Taipei City, Taiwan; ^4^Department of Obstetrics and Gynecology, Chang Gung Medical Center, Taoyuan City, Taiwan; ^5^School of Medicine, Chang Gung University, Taoyuan City, Taiwan

**Keywords:** congenital chylothorax, pneumothorax, hydrops fetalis, EXIT, octreotide

## Abstract

**Background:** Idiopathic congenital chylothorax is a rare but serious disease. Advancement in perinatal care and the renovated treatment modalities have brought about significant improvement in patient outcome.

**Objective:** To describe the clinical course of severe forms of idiopathic congenital chylothorax, focusing on the development of recent treatment modalities and their impacts.

**Design:** A retrospective cohort by review of medical records in the NICU of a perinatal referral center in Taiwan. Study period was from January 2006 to June 2017. Neonates with the diagnosis of idiopathic congenital chylothorax with non-immune hydrops fetalis were enrolled. Clinical relevant including demographic data, perinatal interventions, post-natal course, and treatment outcome were described and analyzed.

**Results:** Twenty-eight neonatal patients were included. The median gestational age at birth was 34 (range 27–36) weeks and median birth weight was 2,369 (range 1,430–3,608) g. Prenatal intervention was performed in 39.3% of the patients. Besides, 11 out of the 28 neonates developed tension pneumothorax in the first 24 h and 4 (36.4%) of them died. Sepsis was documented in two patients (7.1%). Overall survival rate was 71.4%. There were five patients enrolled during the last 2 years of the study period. EXIT with intubation was performed in two and octreotide was given to four of these most recent neonates, and all of them survived.

**Conclusion:** Recent advances in the management of these neonates, specifically EXIT with intubation and use of octreotide. Both of them improved patient survival in our cohort, but the evidence of impact has yet to be validated.

## Introduction

Chylothorax is an accumulation of lymphatic fluid within the pleural space, and congenital chylothorax (CC) is the most common cause of pleural effusion during perinatal period (1, 2). CC can be detected either prenatally or post-natally. It is a rare disease with an estimated prevalence rate of fetal chylothorax of 1 in 15,000 pregnancies, and neonatal chylothorax of 1 in 10,000–24,000 live births, respectively (1, 3, 4). CC has a male:female ratio of 2:1. It can be unilateral, and occurs more often on the right side or bilateral (3, 5). The etiologies of CC are intrathoracic structural anomalies that impede lymphatic drainage, or primary lymphatic disorders (3, 5). Lymphatic disorders could be an isolated event or associated with genetic disorders, such as Turner syndrome, Noonan syndrome, and trisomy 21 (5). In most cases the exact etiology of CC remains undetermined. Clinical consequences associated with CC are mass effect that prenatally results in pulmonary hypoplasia, decreased pre-load, heart failure and non-immune hydrops fetalis (NIHF). After birth, a large amount of pleural fluid may hinder lung expansion and compromise cardiac output, thus put the infant at risk for severe hypoxic ischemic injury. Loss of lymphatic fluid and its components may further result in dehydration, immune-deficiency, coagulopathy, and malnutrition (5). Natural course of CC might vary greatly from being asymptomatic with spontaneous regression, to the development of NIHF. NIHF usually indicates the most critical condition and predict the worst outcome (4, 6, 7).

With the advances in perinatal care, various treatment strategies for CC have been introduced lately. The rationales for prenatal treatment are to alleviate fetal intrathoracic fluid accumulation, so as to allow further lung growth and facilitate cardiac output. Fetal interventions for this purpose include thoracocentesis, placement of pleuro-peritoneal shunts, and pleurodesis (6, 7). Post-natally, the main goal of management is to help lung expansion while allow time for impaired lymphatic drainage to remodel. Nutritional modulation with median chain triglyceride (MCT)-enriched formulae and pharmacotherapy with somatostatin and its analog octreotide, have both been reported to be effective in reducing lymphatic leakage and benefit clinical outcomes in patients with CC (8–11). To minimize duration of possible hypoxia during delivery, extra-uterine intrapartum treatment (EXIT, neonatal management provided upon delivery before the umbilical cord is cut and fetal-placental unit is still functioning) have also been applied in neonates who had prenatally diagnosed severe CC (8). However, due to rarity of the disease and inconclusive reports, there's no consensus in either prenatal or post-natal management of CC.

The aim of this study was to describe our experience in the management of neonates born with idiopathic CC and NIHF, with an emphasis on the impacts of different management strategies on patient outcome.

## Patients and Methods

### Study Design and Patients

This is a case series of patients cared in the neonatal intensive care unit (NICU) of Chang-Gung Memorial hospital, which has a tertiary neonatal referral center for Northern Taiwan. The study was conducted by retrospective review of medical records and was approved by the Institutional Review Board of Chang-Gung Medical Foundation. Patients were identified retrospectively by searching from the neonatal database for all infants admitted to our NICU during the period between January 2006 and June 2017, and had “chylothorax” in their discharge diagnoses. Pleural effusion was detected either prenatally or post-natally by sonogram and/or chest X-ray. Chylothorax was confirmed if the pleural fluid contained more than 110 mg/dL of triglycerides (with enteral feeding), or a total cell count of over 1,000 cells/mL with more than 80% being lymphocytes. In this study we only enrolled neonates of idiopathic CC with NIHF. Patients without hydrops fetalis, or patients of acquired chylothorax (post-operative or traumatic chylothorax), of non-idiopathic chylothorax (with an identifiable cause, such as chromosomal anomalies, structural heart defects, immune mediated diseases, severe anemia, or evidence of viral infection) were excluded.

### Data Collection

Demographic data, time of diagnosis, treatment modalities (*in-utero*, intrapartum, and post-natal) and clinical outcomes were collected and analyzed. Prenatal treatment includes fetal thoracocentesis, pleural-amniotic shunting, and amnioreduction. Intrapartum management (EXIT) includes thoracocentesis with or without endotracheal intubation. Post-natal interventions include drainage of effusion, mechanical ventilation, diet modification, nutritional support and use of octreotide if indicated (such as protracted lymphatic leakage in spite of conventional treatment). Clinical outcomes considered are occurrence of pneumothorax within 24 h, sepsis, and death.

### Statistical Analysis

Data were analyzed using SPSS version 20.0 (SPSS Inc., Chicago, IL, USA.) Mann-Whitney *U* test was used for the comparison of continuous variables and the chi-square test or Fisher's exact test were used for comparison of in-hospital survival. All statistical tests were two-tailed and a *p*-value of <0.05 were considered statistically significant.

## Results

### Patients' Characteristics

**Figure 1:**
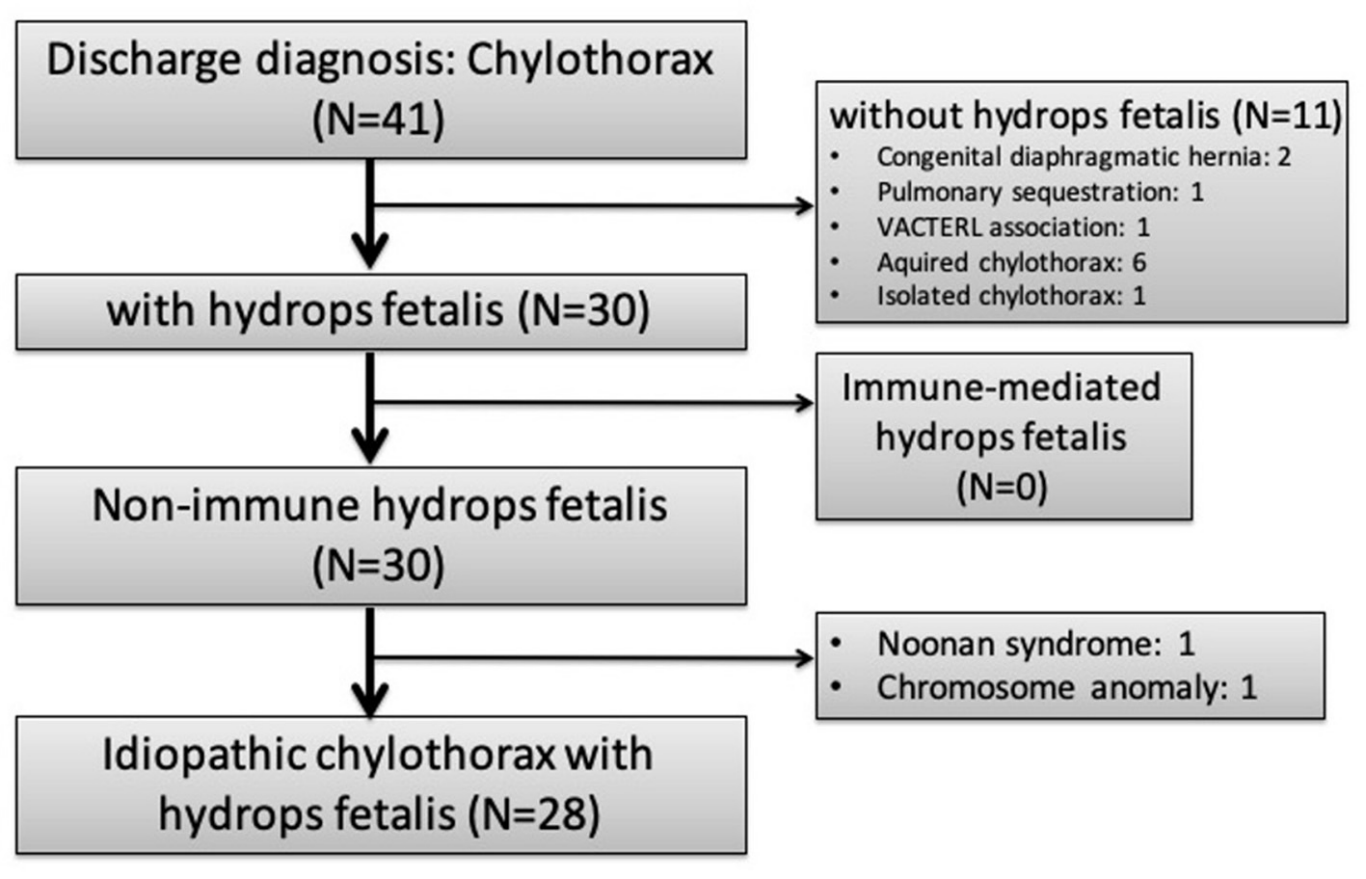
Flow chart of patient enrollment.

During the study period, 41 infants had a discharge diagnosis of “chylothorax” and 28 of them identified as idiopathic CC with NIHF were enrolled (Figure 1). The demographic data of these infants are summarized in Table 1.

**Table 1:** Demographic data and clinical characteristics.

	***N* = 28**
Male (%)	15 (53.6%)
Cesarean section (%)	24 (85.7%)
Gestational age at birth, weeks, Median (range)	34 (27–36)
Birth weight, g, Median (range)	2,369 (1,430–3,608)
Prenatal diagnosis (%)	27 (96.4%)
Gestational age of diagnosis, weeks, Median (range)	31 (18–36)
Apgar score (1 min), Median (range)	4 (0–9)
Apgar score (5 min), Median (range)	7 (1–10)
Bilateral chylothorax (%)	22 (78%)

### Treatment Strategies

As summarized in Table 2, prenatal intervention was performed in 11 patients. Extra-uterine intrapartum treatment (EXIT) was performed in 13 patients, including thoracocentesis alone in 10 and endotracheal intubation followed by thoracocentesis in the last three patients. Ways of nutritional support were consistent in all infants, including delayed enteral feeding and provision of a MCT-enriched formula. The adjunctive pharmacotherapy (octreotide) was given in four infants. None of these patients received pleurodesis for treatment.

**Table 2:** Prenatal, intra-partum, and post-natal interventions.

**Treatment modality**	***N* = 28**
Prenatal intervention (%)	11 (39.3%)
Pleural-amniotic shunt insertion	6
*In utero* thoracocentesis	4
Amniotic fluid reduction	1
Intra-partum treatment (EXIT)	13 (46.4%)
Thoracocentesis only	10
Endotracheal intubation + Thoracocentesis^*^	3
Post-natal management	
Chest tube drainage	24 (86.0%)
Mechanical ventilation	25 (89.3%)
High frequency oscillator	8
Conventional ventilator	17
Octreotide	4 (14.3%)
Starting day of age, median (range)	6 (2–11)
Maximum dose used (mcg/kg/h), median (range)	3.5 (2–8)
Duration of treatment (days), median (range)	17 (14–22)

* *Endotracheal intubation followed by thoracocenteses*.

### Outcomes

The overall survival rate was 71.4%. Early pneumothorax (within 24 h post-natally) occurred in 11 patients, and four of them died shortly afterwards in spite of aggressive resuscitation. These infants accounted for half of the mortalities in the cohort. Six of the 11 patients who developed early pneumothorax received EXIT procedures with thoracocentesis alone. On the other hand, none of those who had EXIT with endotracheal intubation before thoracocentesis developed pneumothorax, and they also all survived. There were two culture proven blood sepsis, and the pathogens were coagulase negative staphylococcus and candida parapsilosis.

We compared the demographics, clinical presentation, and treatment modalities between survivors and non-survivors and presented in Table 3. Higher Apgar scores both at 1 and 5 min in the survivors were the only significant difference when compared to non-survivors (*p* = 0.004). Other prenatal, intrapartum and post-partum factors did not differ between survivors and non-survivors. EXIT with intubation and thoracocentesis was performed in three patients, and all of them survived. Four patients received octreotide all survived.

**Table 3:** Demographics and clinical variables in surviving and non-surviving infants.

	**Survivors**	**Non-survivors**	***p*-value**
	**(*n* = 20)**	**(*n* = 8)**	
**Demographic data**			
Gestational age at birth, weeks	33.5 (30–36)	34 (27–35)	0.795
(median, range)			
Birth weight, g (median, range)	2,248	2,500	0.387
	(1,430–3,520)	(1,471–3,608)	
GA of diagnosis <32 weeks	14	4	0.4004
Male (*n* = 15)	12	3	0.281
Cesarean section (*n* = 24)	16	8	0.172
1-min Apgar score (median, range)	5 (1–9)	1 (0–3)	0.001^*^
5-min Apgar score (median, range)	7.5 (1–10)	4 (1–7)	0.004^*^
**Prenatal factors**			
Gestational age of diagnosis (weeks)	30 ± 4	30 ± 3	0.727
Prenatal therapy (*n* = 11)	8	3	0.904
Bilateral chylothorax (*n* = 21)	14	7	0.569
**Intra-partum factors, EXIT**			
Thoracocentesis only (*n* = 10)	7	3	1
ET + Thoracocentesis (*n* = 3)^#^	3	0	0.536
**Post-natal factors**			
Pneumothorax within 24 h (*n* = 11)	7	4	0.672
Culture proven sepsis (*n* = 2)	1	1	0.497
Octreotide (*n* = 4)	4	0	0.295

* *p < 0.05*.

# *ET + Thoracocenteses = endotracheal intubation followed by thoracocentesis*.

Among the survivors (*n* = 20), 17 had persistent post-natal chylous pleural drainage that lasted for a mean duration of 17.2 ± 9.2 (Mean ± S.D.) days. Enteral feeding was initiated at a mean age of 4.5 ± 2.8 days, and was fully established after 16.6 ± 14.1 days. Duration of mechanical ventilator was 28.6 ± 21.0 days, and the mean length of hospital stay was 47.5 ± 24.9 days.

## Discussion

In this retrospective review we included 28 infants diagnosed idiopathic CC with NIHF. We described the stepwise treatment modalities that evolved with time, and observed an ever improving patient survival rate reaching 100% lately. Our overall survival rate over the study period was 71.4%, compared to 30–70% in recent report (5). Even though spontaneous resolution may occur, those who develop hydrops fetalis represents the most severe conditions, and hydropic change has also been identified as the single most important prognostic factor of a poor outcome in CC (8). Our report of this invaluable cohort will provide the much-needed insights and fill in the knowledge gap for how to better manage patients with severe CC.

**Figure 2:**
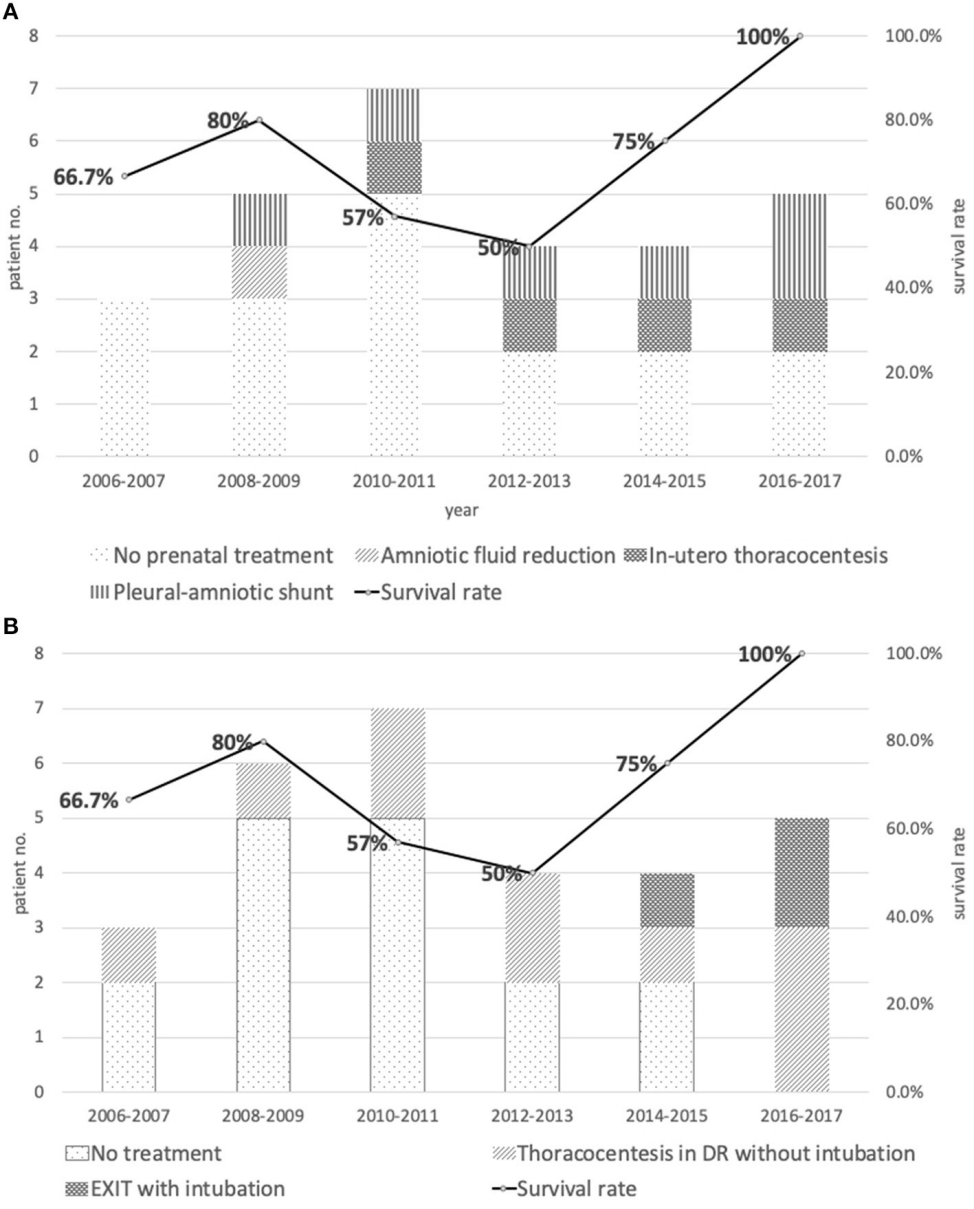
**(A)** Distribution and types of prenatal treatments vs. survival rate between 2006 and 2017. **(B)** Distribution and types of intrapartum management vs. survival rate between 2006 and 2017.

Different types of prenatal interventions have been developed to reduce intrathoracic fluid and to allow continuous fetal lung development and prevent circulatory embarrassment. Petres et al. reported the first *in-utero* thoracocentesis in 1982 (9), and Roberts et al. described insertion of a thoraco-amniotic shunt for successful prenatal pleural drainage and reverse the progression of fetal hydrops in 1986 (10). So far conclusions on the safety, efficacy, and indication for prenatal drainage of fetal chylothorax has not been reached, except the consensus among specialists that invasive *in-utero* therapy for fetal hydrothorax should be reserved for hydropic fetuses without additional anomalies ((12)). In an earlier systematic review Deurloo et al. found no significant differences in mortality rates using different therapies of single thoracocentesis, multiple thoracocentesis, thoracoamniotic shunting or combined thoracocentesis and shunting as prenatal treatment options for isolated fetal chylothorax (11). In our series, only 11 patients (39.3%) received prenatal treatment and the treatment modalities included initially amnio-reduction in one patient, fetal thoracocentesis in four, and more recently thoracoamniotic shunting in six patients. As seen in Figure 2, our progress in the fetal intervention did bring about improved treatment results. However, in this cohort overall prenatal treatment did not affect patient survival. Due to relatively small case number and the variation in fetal conditions at the time of referral, neither conclusion on benefit of treatment nor comparison of efficacy among different treatment modalities could be justified from this study.

Other prenatal factors that might influence neonatal survival in infants with CC included gestational age of diagnosis, treatment-to-delivery interval, refractory hydrops regardless of fetal drainage, and degree of prematurity (11, 13, 14). Outcome has been worse when patients were diagnosed at a lower gestational age and when effusions were bilateral in some reports (5, 15). In our study, there was no significant difference in gestational age of birth between survivors and non-survivors. However, all of our non-survivors were diagnosed prenatally, and most of them (7 out of 8) were diagnosed before 32 weeks of gestation. This is consistent with previous study showing CC detected before 32 weeks of gestation usually has a poor prognosis, and if patients develops hydrops fetalis, mortality rate is almost 100% (16). These seven non-survivors in our study were also delivered relatively less immature, after 34 weeks of gestation. We reasoned that these babies might have been severely compromised at the time of referral, which resulted in their death even though they were born with a lower degree of prematurity.

EXIT was applied first in patients with prenatally diagnosed congenital diaphragmatic hernia (17), and later as part of the perinatal management of patients with congenital obstructive malformations of upper airways (18). EXIT procedures help to gain time for safe adaptation of newborn to the *ex-utero* life, by establishing an intact airway for the post-natal lung to ventilate. Prontera et al. first applied EXIT procedure in a case with severe bilateral fetal pleural effusion. The team performed bilateral thoracocentesis to evacuate pleural fluid on the operating tables before the cord was cut, aiming to leave enough space for lung expansion when the infants initiates effective spontaneous breathing. Later on, the infant was separated from the feto-placental unit and brough to a radiant warmer for endotracheal intubation and resuscitation (19). Initially we adapted this EXIT procedure of intra-partum thoracocentesis as well. However, in our experience, thoracocentesis alone does not guarantee the patient a smooth passage to *ex-utero* life. Instead, early pneumothorax often happened to those who received EXIT with thoracocentesis alone. It has been reported that live-born infants with CC who developed early pneumothoraces usually had worsened outcome (20), and our patients are no exceptions. Reviewing the cause of death in our cohort, six of the eight mortality patients died of respiratory failure, including four caused by tension pneumothorax immediately after birth, all received EXIT with thoracocentesis alone. As for the cause of pneumothorax, there are certain obvious iatrogenic candidates which also involved pulmonary hypoplasia and high ventilator pressure. However, we speculated that the pathophysiology of pneumothorax after thoracocentesis in these infants might involve so called “pneumothorax ex vacuo.” Pneumothorax ex vacuo occurs if the lung is unable to expand and thoracocentesis in this situation generates a low intrapleural pressure which transiently opens a tiny hole in the lung to allow air into the pleural space (alleviating the “vacuum”) (21). The risk of pneumothorax ex vacuo also includes rapid removal of large amount of pleural fluid (22). This reasonably explains why in the case report of Eraslan, their EXIT procedure (containing thoracocentesis alone) in an infant with unilateral fetal hydrothorax inadvertently resulted in contralateral pneumothorax (16). We later modified our EXIT procedures as follows: sequentially, endotracheal intubation, gentle manual ventilation, and needle aspiration of moderate amount of fluid just sufficient for lung expansion, before cutting the cord. The last three patients managed by this modified EXIT procedure all had smooth post-natal transition and survived with intact neurological outcome.

Latest advances in the management of CC also include use of octreotide. Somatostatin was first used for treating infantile chylothorax in 1998 (23). Mechanism for the pharmacological effect relies on its blockage of the growth hormone release, and depressed insulin and lymph fluid excretion by reducing splanchnic blood flow and intestinal secretion of electrolytes and water (24). Octreotide is a synthetic analog of somatostatin with a longer half-life. Most studies of octreotide use for CC were case reports or small case series. Even in the earlier systemic reviews of somatostatin/octreotide use in young infants or neonates did not reach the same conclusion: Roehr et al. found obvious treatment efficacy in young infants with primary or secondary chylothorax with various doses and treatment duration, whereas Das and Shah concluded that routine use of octreotide for neonates with CC could not be recommended owing to limited number of cases and variable dose and administration schedule in the review (25, 26). Very recently, Yin reported that somatostatin/octreotide treatment reduced pleural drainage and helped lowering ventilator support without significant side effects in neonates with CC (27). Four of our patients received octreotide at a median maximal dose of 3.5 mcg/kg/h and duration of 17 days. All of these treated infants survived without significant side effects. Our experience was in consistence with the study by Bellini et al. in which they found octreotide, in a median initial dose of 2 mcg/kg/h, a median maximum dose of 7.5 mcg/kg/h, and a median treatment duration of 16 days, to be a relatively effective and safe treatment option in neonates with chylothorax, especially for the congenital forms (28).

We reported our accumulated experience of managing the rare and critical condition of CC with NIHF for more than a decade. Along with the comprehensive prenatal, intrapartum, and post-natal management in recent years, we seemed to have better result in patient outcome. Specifically, the survival improved markedly in these neonates after our modified EXIT procedures and post-natal octreotide in those with refractory chylous drainage.

The limitation of this study is that it is a single institute, non-randomized retrospective study without long-term follow-up. Those patients of intrauterine death or spontaneous resolution before delivery were not accounted for, and there was no classification of the degree of disease severity at patients' entry of the study.

## Conclusion

Patients with prenatally diagnosed hydrothorax should be referred urgently to a tertiary center with experience in intensive fetal treatment modalities and meticulous perinatal care including intrapartum therapy. With adequate treatment, the majority of the fetuses with CC are likely to survive, even in those most severe ones with NIHF. Our experience could provide insights into fetal and neonatal responses to various treatment modalities for this disease entity, and aid to future development of the much-needed treatment guidelines.

## Data Availability Statement

The data that support the findings of this study are available from the corresponding author, Reyin Lien, upon reasonable request.

## Ethics Statement

The studies involving human participants were reviewed and approved by Institutional Review Board (IRB) of Chang-Gung Medical Foundation deemed this study exempt (Number 201800602B0). Written informed consent for participation was not provided by the participants' legal guardians/next of kin because: it's a retrospective study with chart review.

## Author Contributions

H-LT conceived the study. H-LT and TM participated in data collection and drafted the manuscript. H-LT, TM, A-SC, S-MC, and RL participated in its design, analysis and interpretation of data, and coordination. RL critically revised the manuscript for important intellectual content. All authors participated in final approval of the version to be published and agreed with all aspects of the work in ensuring that questions related to the accuracy or integrity of any part of the work are appropriately investigated and resolved.

## Conflict of Interest

The authors declare that the research was conducted in the absence of any commercial or financial relationships that could be construed as a potential conflict of interest.
